# An Overview of Hot Carrier Degradation on Gate-All-Around Nanosheet Transistors

**DOI:** 10.3390/mi16030311

**Published:** 2025-03-06

**Authors:** Huimei Zhou

**Affiliations:** IBM Research Albany, 257 Fuller Road, Albany, NY 12203, USA; zhouhuim@us.ibm.com

**Keywords:** gate-all-around, nanosheet, transistor scaling, reliability, hot carrier degradation, self-heating, corner effects, inner spacer, junction

## Abstract

Gate-All-Around (GAA) Nanosheet (NS) transistors have been identified as the device architecture for 3 nm and beyond as they provide additional scaling benefits. The Hot Carrier (HC) effect cannot be ignored in the development of metal oxide semiconductor field effect transistors (MOSFETs). In this article, we present a comprehensive review of Hot Carrier Degradation (HCD) studies on GAA NS transistors including geometry dependencies, surface orientation impacts, corner effects, characterization methodologies, process impacts and self-heating impacts from different researchers, together with the challenges and outlook, providing an insightful and valuable HCD reliability discussion and review on the cutting-edge technology in continuous MOSFET scaling.

## 1. Introduction

Transistors have scaled down for decades to achieve lower power consumption, higher density and higher speed. Short channel effects (SCEs) become increasingly serious as the channel length is scaled down [1,2,3,4]. Better electrostatic control of the channel by the gate is required to suppress short channel effects. Vertical stacked GAA nanowire/nanosheet structures can offer excellent electrostatics and good short channel control, enabling continued transistor scaling. GAA MSOFETs provide design solutions at 3 nm technology nodes and beyond [1,2,3,4]. The International Roadmap for Devices and Systems (IRDS) predicted the roadmap of logic transistor for the next generations, indicating that GAA Field Effect Transistors (FETs) were selected for the transistor structures beyond FinFET in 2020 [5]. Furthermore, NS technology has been announced towards mass production from different providers [6,7]. Many researchers have worked on GAA NS transistors and reported in-depth studies on performance as well as reliability to optimize and explore mechanisms specific to the new transistor architecture [8,9,10]. 

Hot carrier effect (HCE), as one of the key mechanisms of reliability, can’t be ignored in the development of metal oxide semiconductor (MOS) FETs, especially n-type MOSFETs [6,7,8,9,10]. Various circuit applications are significantly limited by HCD induced threshold voltage (V_th_) shift, drive current (I_ON_) reduction, Subthreshold Slope (SS) degradation, and transconductance (G_m_) degradation [11,12]. GAA NS transistors provide the best control of SCE allowing channel length scaling disproportionate to the operating voltage. The degradation caused by hot carriers (HCs) in highly scaled GAA NS transistors must be studied as extensively as other subjects [13] and a thorough review of the HCD mechanism specific to GAA NS architecture becomes imperative. 

In this article, a comprehensive review on HCD studies of GAA NS transistors is performed to give the readers an in-depth understanding of the HCD reliability mechanism of NS transistors as well as specific components of GAA structure. The paper is structured as follows: we begin in Section 2.1 with GAA NS structure and gate length scaling, by providing an illustration of a typical GAA NS architecture with key components including STI, inner spacer, etc. In Section 2.2, a discussion of HCD characterization methodology and HCD impact on device parametric and trap generation during stress of different V_GS_/V_DS_ combinations is presented. In the next three Section 2.3, Section 2.4 and Section 2.5, we review HCD due to corner effects, geometry effects, surface orientation and process impact of GAA NS transistors. As NS specific components, dielectric wall and substrate isolation, whose impacts on HCD of NS transistors are compared with their reference in Section 2.6 and Section 2.7. Self-heating studies are discussed in Section 2.8 with some pioneering studies on HCD reliability qualification. Finally, challenges are discussed from current research works in Section 3 together with outlooks of future research directions towards continuous scaling down.

## 2. Structure, HCD Characterization Results, and Discussion

### 2.1. GAA NS Structures and Gate Length Scaling

**Figure 1:**
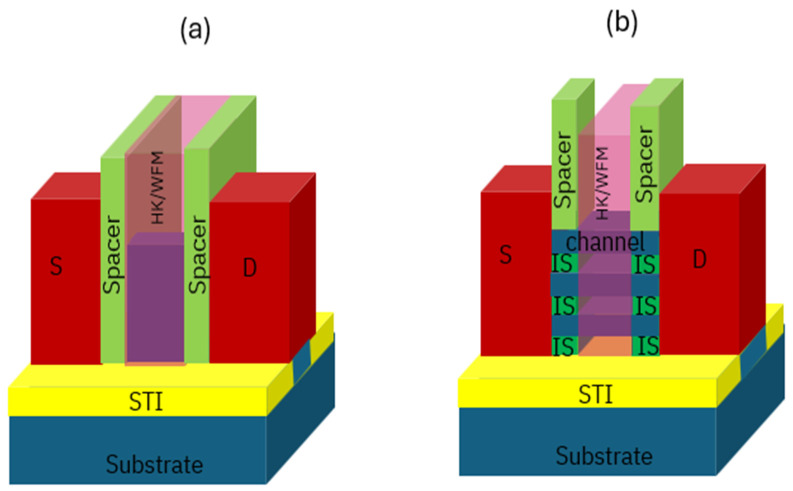
Schematic diagram of (**a**) a FinFET and (**b**) a GAA nanosheet transistor respectively. Fins/Nanosheets horizontally isolated by shallow trench isolation (STI) from bottom. Source/drain (S/D) epitaxy, and a high-k (HK), work function metal (WFM) metal are marked and plotted in the Figure. Gate length could be further scaled with gate wrapping round the nanosheets for excellent short channel control.

FinFETs architecture has substituted planar FETs as mainstream logic transistor structure for numerous generations. GAA architecture was invented and developed to meet logic device scaling needs by offering excellent short channel control as a replacement of FinFETs on 3 nm technology and beyond. Figure 1 shows the schematics of (a) a FinFET and (b) a bulk GAA NS FET with three vertically stacked Si channels. Key components of the two architectures are highlighted in the figures. Fins/Nanosheets, plotted in dark blue color in the figures, are horizontally isolated by shallow trench isolation (STI) from the bottom substrate, and the source/drain (S/D) epitaxy and a High-K (HK)/Work Function Metal (WFM) are marked and plotted in red color and pink color in the Figure Spacer, plotted in light green color, isolates the S/D and gate in both the FinFET and GAA NS transistors. Inner spacer (IS), as a special structure in GAA NS, is marked and plotted in Figure 1b in dark green color. It is observed that Fins are wrapped on three sides (Tri-gate) by HK-Metal gate in. Figure 1a, nanosheets are completely wrapped by HK-Metal gate in Figure 1b. In GAA NS architecture, multiple sheets are stacked on top of each other to achieve high current and performance benefit without sacrificing footprint [12,13]. As one of the key benefits in scaling, gate length can be further scaled with good short channel control, attributing from nanosheets being fully wrapped by HK/WFM gate.

**Figure 2:**
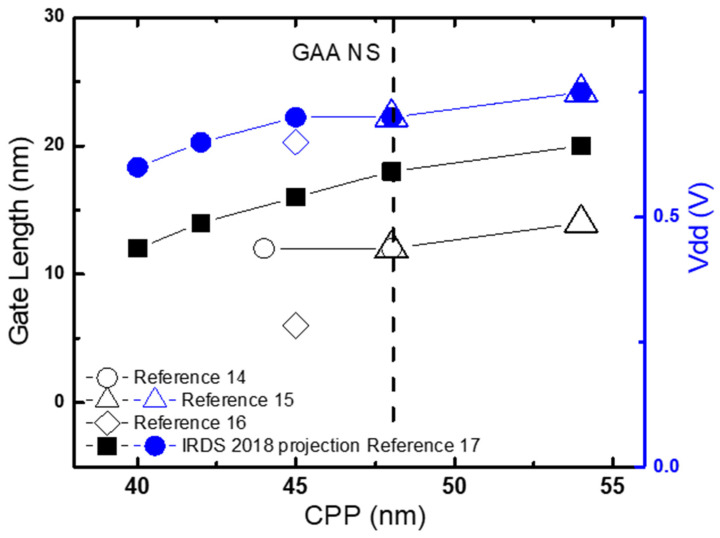
Gate length in Nanometer (nm) and operation voltage in Volts (V) vs. Gate Pitch (CPP) in nm for transistors from [14,15,16,17]. Device architecture was changed to GAA NS at 54CPP and below. Gate pitch, gate length and operation voltages are scaled down with transistors size shrinking.

Logic transistors density scaling is achieved by gate pitch (CPP) scaling in one direction and transistor width/space scaling in the other direction. Figure 2 shows the trend of gate length and operation voltage trends vs. gate pitch for transistors from publications and IRDS projection [14,15,16,17]. Device architecture was changed to GAA structure at 54CPP and below. Gate pitch, gate length and operation voltage were all scaled with transistor scaling.

**Figure 3:**
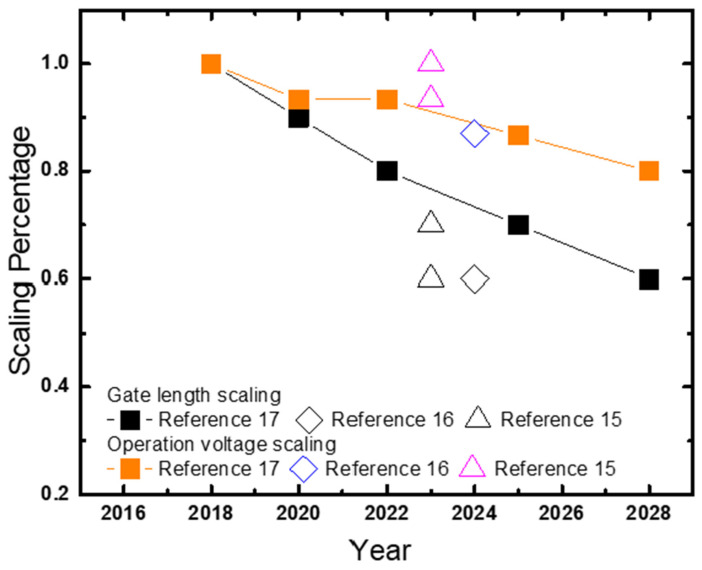
Scaling percentage vs. Year for gate length and operation voltages for transistors from [14,15,16,17]. Operation bias scaling is observed to be less than dimensional scaling.

Figure 3 shows scaling in the form of percentage vs. year for gate length and operation voltage of logic transistors from publications and IRDS projection [14,15,16,17]. The operation bias does not decrease as fast as gate length scaling resulting in transistors operated at higher electric field compared to previous generations. HCD has become more concerning and is now an important reliability mechanism in GAA NS transistors, especially n-type FETs, due to aggressive gate length scaling.

### 2.2. HCD Characterization

**Figure 4:**
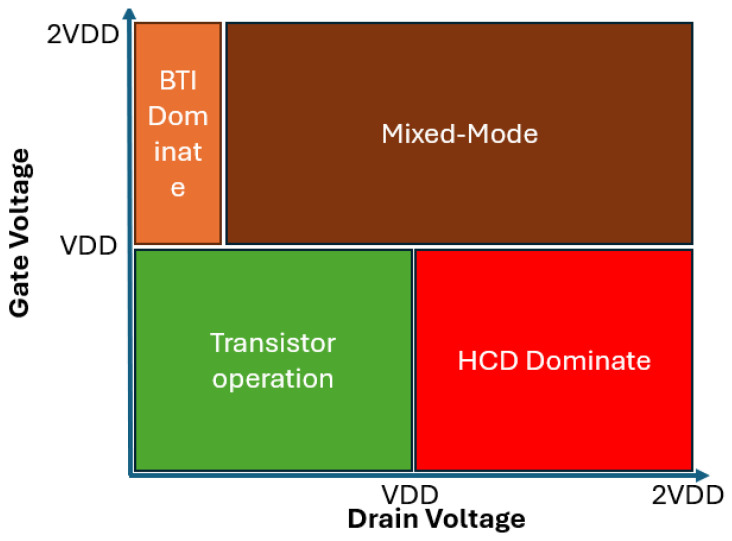
Schematic diagram of different V_GS_, V_DS_ bias together with various degradation mode. VDD represents transistor operation voltage. Pure HCD dominating region occurs under the stress condition of high V_DS_ and low V_GS_ in red color zone. Reliability stress typically was performed at stress voltage lower than 2VDD.

Device parametric shift such as ∆I_dlin_, ∆I_dsat_, ∆V_th_, ∆G_m_ etc. are generally used in HCD studies to monitor and project end-of-life performance of transistors and circuits. HCD has been observed to be worst at high V_GS_ and V_DS_ conditions (such as V_GS_ ≈ V_DS_ > VDD) in 20 nm technology node and beyond. The degradation mode, driven by the current density, becomes dominant [18,19,20]. Schematic plot of different V_GS_, V_DS_ bias together with the various degradation modes is shown in Figure 4. Transistors typically operates in the block area with green color, voltage accelerate characterization is performed and modeled to project End-of-Life (EOL) performance of transistors and circuits. Under high V_GS_ and extremely low V_DS_ stress, Bias Temperature Instability (BTI) dominates the degradation as shown with the block with the orange color. Under high V_DS_ and low V_GS_ stress, hot carrier dominates the degradation, as shown with the red color block. Similar illustration of reliability dominating mechanism with different V_GS_/V_DS_ configuration was also reported in [20]. Various transistor measurement methodologies were developed to isolate/de-couple BTI, HCD and self-heating [18,19,20,21,22,23,24].

Zhou et al. reported HCD study under V_GS_ < V_DS_ and V_GS_ = V_DS_ stress on scaled gate length of GAA NS n-type transistors with self-aligned substrate isolation at various temperatures [21]. ∆I_dsat_ was used to identify the degradation during hot carrier stress, device parametric shift of V_t_ and G_m_ were studied and correlated to I_dsat_ degradation post stress. ∆V_t_ vs. ∆I_dsat_% and ∆G_m_/G_m0_ vs. ∆I_dsat_% were reported showing reversed trend post Mid-V_GS_ and High-V_GS_ stress, indicating different oxide/interface traps combination accumulated post stress, corresponding to various device parametric response [21]. TCAD simulation was also performed in the article to decouple interface traps and oxide traps based on device parametric shift before and post Mid-V_g_ and High-V_g_ HCI stress. It’s observed that post Mid-V_G_ HCI stress, HCD caused I_dsat_ degradation occupy more percentage from interface traps. Post High-V_g_ HCI stress, the transistor operates at mixed mode as illustrated in Figure 4, oxide traps occupy more percentage of the total I_dsat_ degradation.

**Figure 5:**
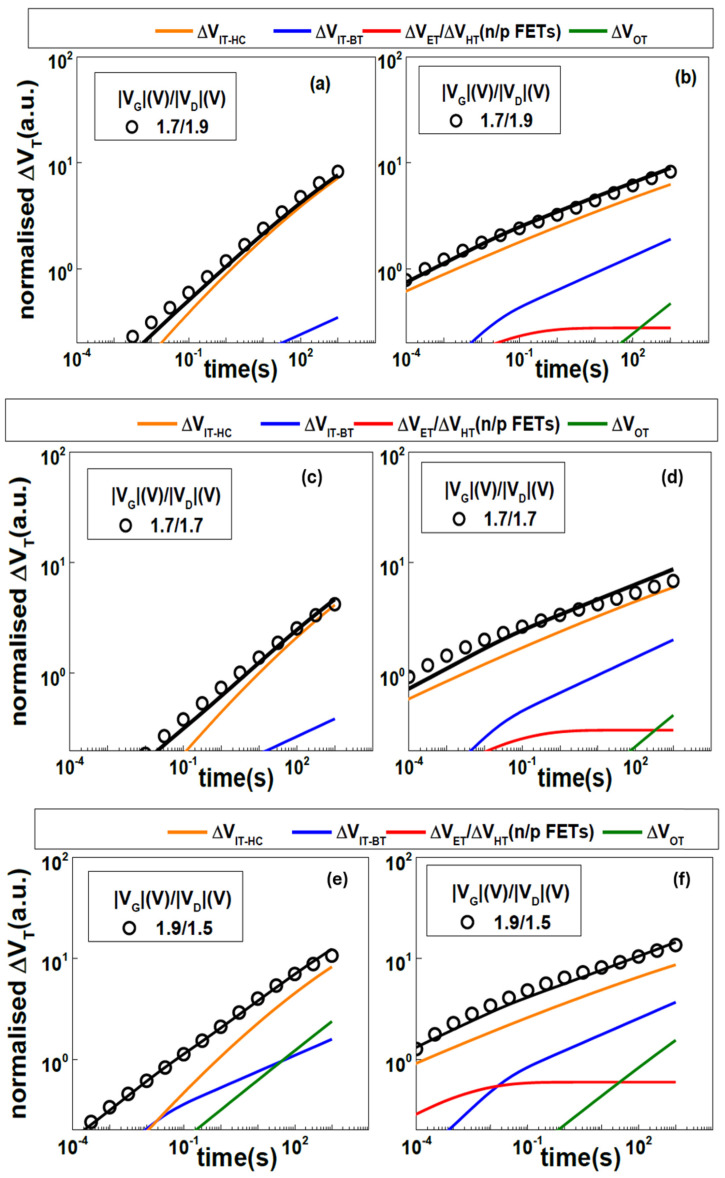
Measured (circles) and modeled (lines) ΔV_T_ vs. stress time for (**a**,**c**,**e**) n-type and (**b**,**d**,**f**) p-type GAA NS transistors under various V_GS_/V_DS_ stress condition at room temperature together with the various subcomponents [22]. Oxide traps caused ΔV_t_ increase with V_GS_ increasing. Reprinted/adapted with permission from IEEE Proceedings of the 2020 International Reliability Physics Symposium.

Threshold voltage shift (∆V_T_) was used to evaluate HCD stress in n-type and p-type channel L_g_ = 60 nm bulk GAA NS transistors at room temperature, reported by Choudhury et al. in [22]. Figure 5 shows measured and modeled normalized ∆V_T_ under different HCD stress conditions. It’s observed that oxide traps increased when V_GS_ stress increase, which is consistent with the observation from [21].

**Figure 6:**
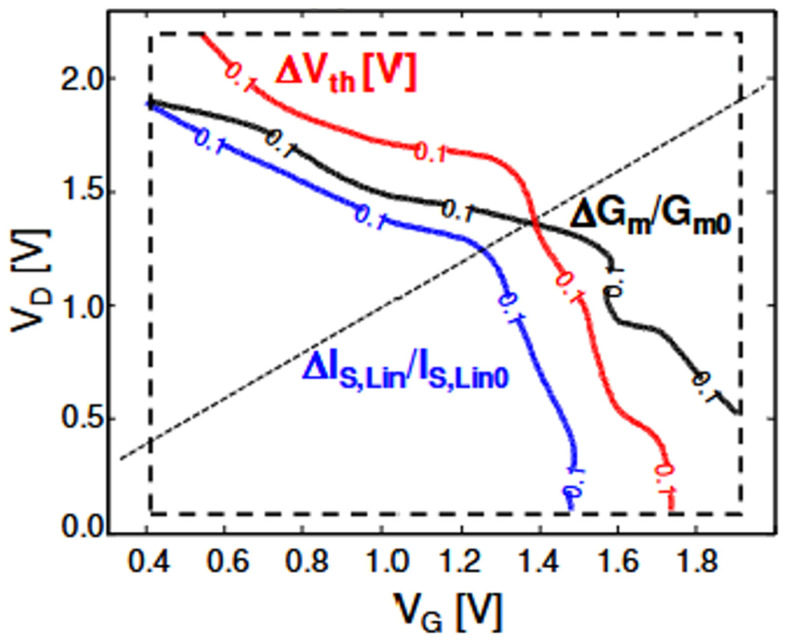
Contour plot at 10% degradation of I_Slin_, G_m_, and ΔV_TH_ = 0.1 V. To achieving 10% G_m_ degradation, lower V_DS_ stress is required comparing with the stress condition of achieving 0.1 V ∆Vth degradation at low V_GS_ and high V_DS_ stress, while at low V_DS_ and high V_GS_ stress, to achieving 10% G_m_ degradation, more V_DS_ stress is required comparing with reaching 0.1 V ∆Vth degradation [23]. Reprinted/adapted with permission from IEEE Proceedings of the 2017 International Electron Device Meeting.

Chasin et al. also studied the effects of HCD under different stress by analyzing the experimental data in n-type L_g_ = 28 nm bulk GAA nanowire transistors at 125 °C under full V_Gs_, V_DS_ bias condition [23]. Figure 6 shows contour plot at 10% degradation of I_Slin_, G_m_, and ΔV_th_ = 0.1 V under various V_GS_ and V_DS_ stress. In high V_DS_ and low V_GS_ stress, to achieving 10% G_m_ degradation, lower V_DS_ stress is required comparing with the stress condition of achieving 0.1 V V_th_ degradation. While in low V_DS_ and high V_GS_ stress, higher V_DS_ stress is required when comparing the stress condition of achieving 10% degradation of G_m_ and 0.1 V V_th_ shift. The cross of the two parameters (ΔV_th_ and ΔG_m_/G_m_) in the contour plot indicates the response of the device parametric changes at low V_GS_/high V_DS_ and high V_GS_/low V_DS_ stress condition, which was also reported by Zhou et al. in [21] regarding slope change of ∆V_t_ vs. ∆I_dsat_ and ∆G_m_ vs. ∆I_dsat_ under Mid-V_GS_ and High-V_GS_ stress. 

### 2.3. Corner Effects

3D transistors are a key breakthrough of device architecture in semiconductor manufacture, allowing transistors further scaling down. The specificities of the 3D architecture such as GAA NS are always associate with complexity processes. Corners of different transistors were observed critical to technology success, device performance, overall uniformity and full wafer yield [25,26]. Corner effects, an existing topic in planar FET and FinFET, continue to be one of the important factors and contribute to new issues in GAA NS architecture, attracting more researchers’ attention and stimulating numerous reliability learnings.

**Figure 7:**
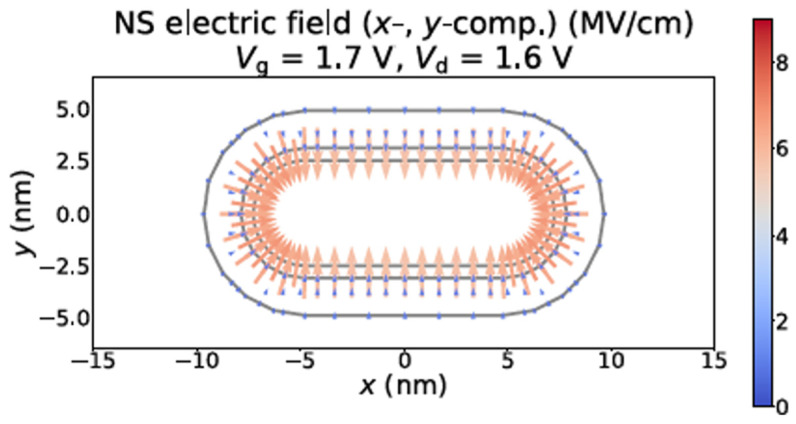
Simulation of oxide electric field at 4 nm from the drain of NS transistor. Oxide electric field and the carrier concentration were observed higher in the curved region comparing to the one in the flat regions [24]. Reprinted/adapted with permission from IEEE Proceedings of the 2022 International Reliability Physics Symposium.

Simulation was performed and reported by Vandemaele et. al on corner effect of GAA NS transistors in [24]. Figure 7 shows simulation of oxide electric field at 4 nm from the drain of NS transistor. It’s found that in the curved region, oxide electric field is higher than the one in the flat regions, which suggests higher interface traps generated during stress in the curved cross section parts comparing with the one generated in flat region, attributed to aggravated reliability [24].

**Figure 8:**
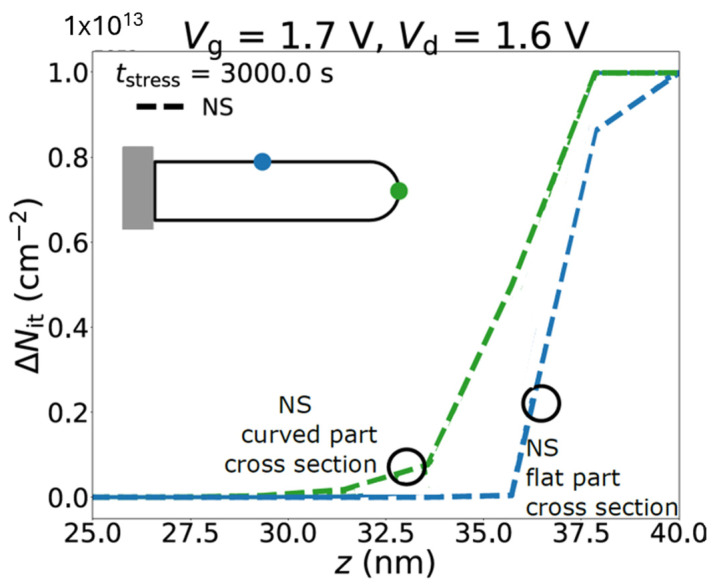
Interface traps density profiles for NS at different positions along the cross section during stress. The curved part of the cross section shows higher interface traps during stress comparing to the one generated at flat part [24]. Reprinted/adapted with permission from IEEE Proceedings of the 2022 International Reliability Physics Symposium.

Interface traps density profiles during stress for NS at different positions along the cross section at certain stress time is shown in Figure 8. Higher interface traps density was observed in the curved part of the cross section compared to the one in the flat region, indicating more interface traps were generated during stress at curved region. As Vandemaele et. al concluded in the paper, HCD was observed to be worse on 3D transistor structures with more curved shapes [24].

**Figure 9:**
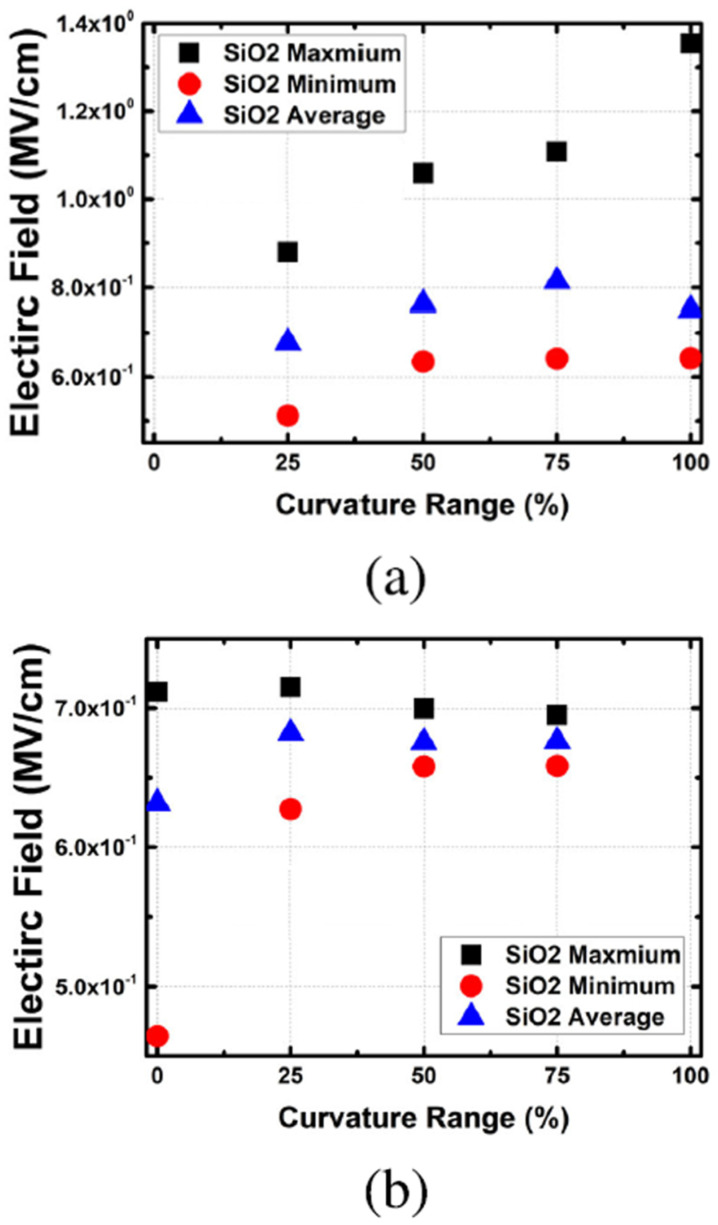
(**a**) Electric field of SiO_2_ at curved edge (**b**) Electric field of SiO2 in the edge flat region with different curvature range [26]. Reprinted/adapted from [27], under a Creative Commons Attribution-NonCommercial-NoDerivatives 4.0 License. Source: https://creativecommons.org/licenses/by-nc-nd/4.0/, accessed on 30 December 2024. Modifications were made to the original Figure.

Curvature range was studied by Lim et al. in [27]. Figure 9a shows the electric field of SiO_2_ with different curvature range at curved edge and Figure 9b shows the electric field of SiO_2_ with different curvature range at flat area. It’s observed that the electric field was higher and increased with curvature range, indicating curvature area of SiO_2_ would suffer higher electric field during stress comparing with flat area, which aggravated reliability [27]. 

**Figure 10:**
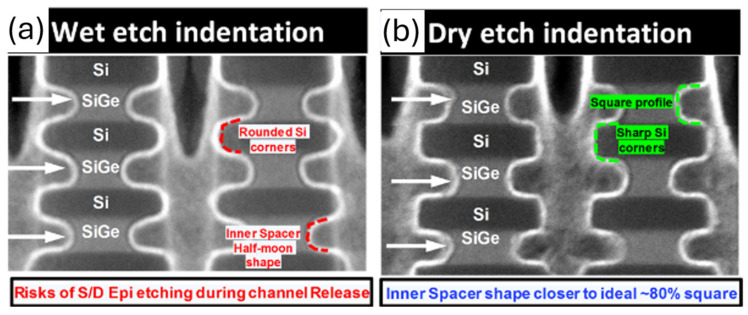
TEM images of (**a**) wet and (**b**) dry etch sacrificial SiGe remove process [28]. It was observed that sheet curvature range could be different from different process. Reprinted/adapted with permission from IEEE Proceedings of the 2019 International Electron Device Meeting.

Different shapes, curvature of nanosheets could be adjusted by various process and design. Figure 10 shows TEM images of nanosheet under different SiGe remove process. Ellipse-shaped sheets was found in Figure 10a and rectangular-shaped sheets was observed in Figure 10b, reported by Loubt et al. in [28]. The paper also reported higher performance from the rectangular shape, which indicated rectangular shape nanosheet not only improved reliability, but also benefited transistors’ performance.

### 2.4. Geometry (W_si_, T_si_) and Surface Orientation Effect

**Figure 11:**
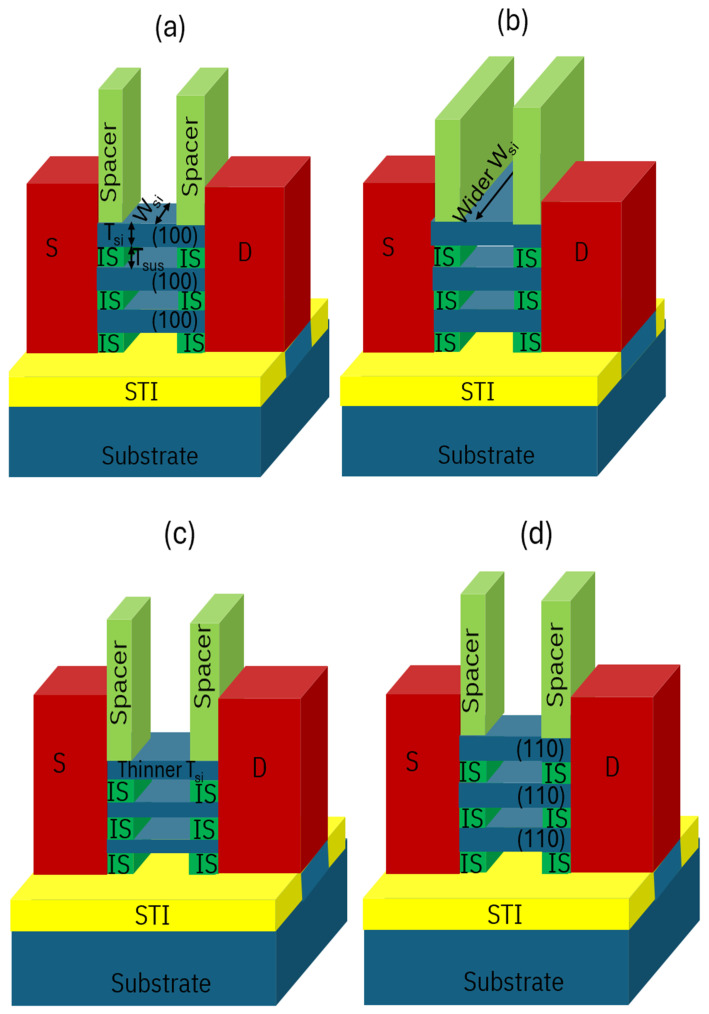
Schematic diagram of (**a**) a NS transistor with typical sheet width (W_si_,), sheet thickness (T_si_), sheet to sheet space (T_sus_) and (100) surface transportation, (**b**) a NS transistor with wider W_si_, (**c**) a NS transistor with thinner T_si_ with the same sheet to sheet space (T_sus_) and (**d**) a NS transistor with (110) surface orientation. Sheet width (perpendicular to gate) increases in (**b**); sheet thickness decreases with the same T_sus_, shown in (**c**); surface transportation changes to (110) orientation from epitaxy growth on Si substrate, shown in (**d**).

The 3D architecture specific components of nanosheet width and thickness coupled with design, process and transportation orientation have led to lots of research on device performance and reliability [29,30]. As one of the benefits of GAA architecture, various sheet width is applicable for different applications. Sheet thickness and surface orientation were also explored for further scaling and pFET performance boost [31,32]. Figure 11 shows schematic diagram of (a) a GAA NS transistor with typical sheet width (W_si_,), sheet thickness (T_si_) and (100) surface transportation, (b) a GAA NS transistor with wider W_si_, (c) a GAA NS transistor with thinner T_si_ with the same sheet to sheet space (T_sus_) and (d) a GAA NS transistor with (110) surface orientation.

Slightly degraded Negative Bias Temperature Instability (NBTI) reliability on GAA NS transistors with narrower W_si_, T_si_ and (110) surface orientation was reported by Wang et al. and Zhou et al. in [33,34,35], attributed to contribution of flat and curved region on NS, together with combination of various surface orientation from surface and sidewall of NS. HCD on sheet width dependance of GAA NS transistors were also further studied and reported by Wang in reference [35,36]. 

**Figure 12:**
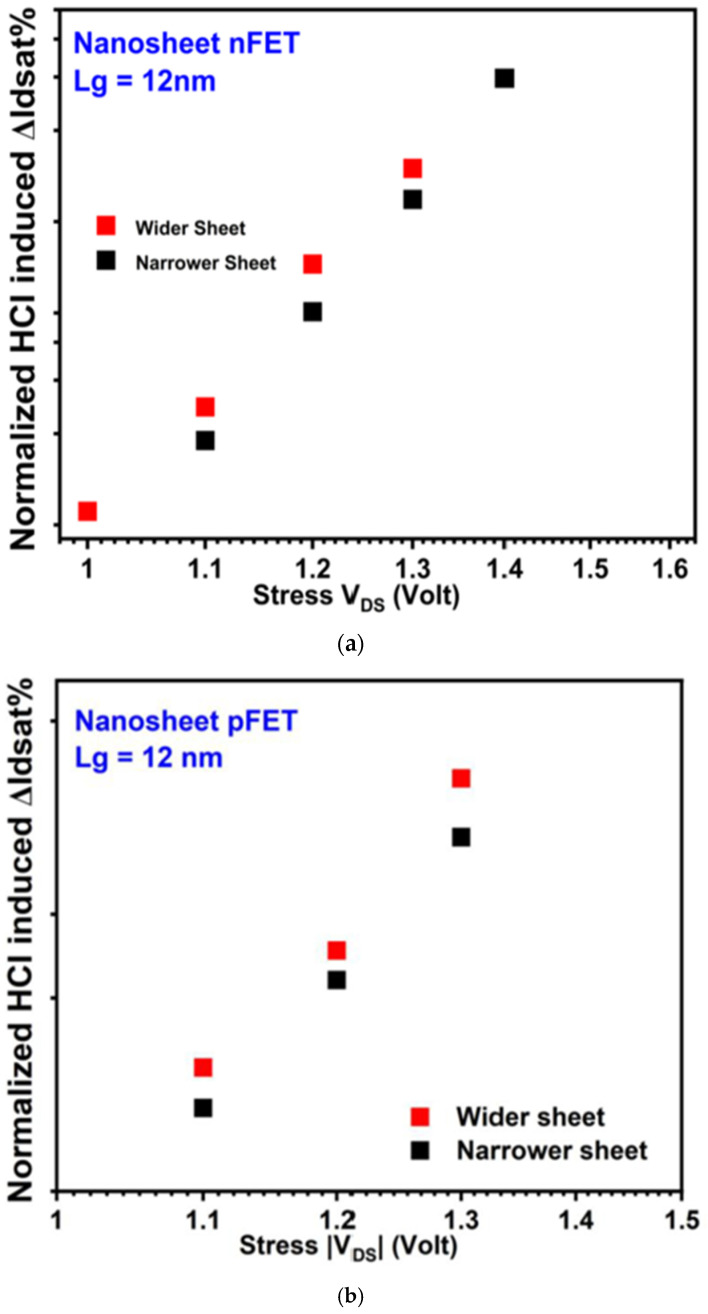
HCD of GAA NS (**a**) n-type and (**b**) p-type transistors with narrow and wide sheet width. GAA NS transistors with wider sheet width show degraded HCD [35]. Under a Creative Commons CC by 4.0 License. Source: https://www.mdpi.com/authors/rights/, accessed on 30 December 2024. Modifications were made to the original Figure.

Figure 12 shows HCD of GAA NS (a) n-type and (b) p-type transistors with narrow and wide sheet widths. Opposite to the results of BTI improving on GAA NS transistors with wider W_si_, HCD was found slightly worse on both n-type and p-type GAA NS transistors with wider sheet width [35]. Root cause of W_si_ dependance of HCD on GAA NS transistors was discussed in the paper. Although interface traps at curve edge region occupy less percentage in wider sheet, more intense self-heating effect from higher current in wider sheets contributed to operation current decreasing, resulting in worse HCD in wider sheets [35]. 

**Figure 13:**
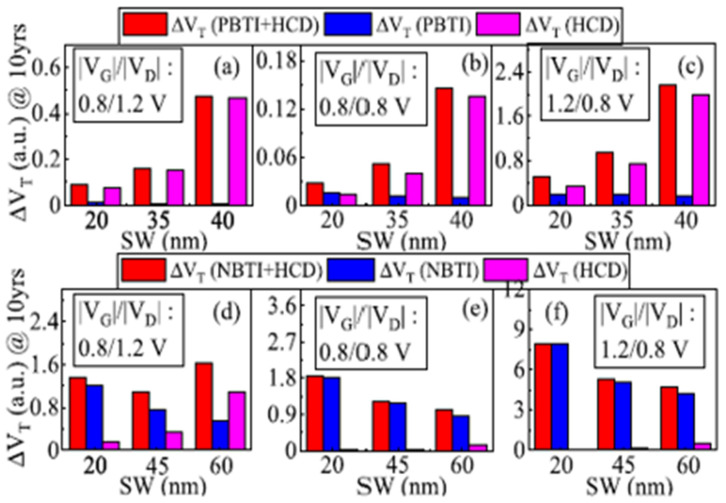
Projected EOL ΔVT vs. different sheet width at various V_GS_ and V_DS_ combination on (**a**–**c**) n-type GAA NS transistors and (**d**–**f**) p-type GAA NS transistors [37]. Reprinted/adapted with permission from IEEE TRANSACTIONS ON ELECTRON DEVICES 2022.

Figure 13 shows projected EOL ΔV_T_ vs. different sheet width at V_GS_ < V_DS_, V_GS_ = V_DS_ and V_GS_ > V_DS_ stress conditions on (a), (b) and (c) n-type GAA NS transistors and (d), (e) and (f) p-type GAA NS transistors, reported by Choudhury et al. in [37]. Consistent with the results in [36,38], HCD was observed to degrade with sheet width increasing at low V_GS_ (V_GS_ < V_DS_) stress in both n-type and p-type GAA NS while comparable or slightly worse under V_GS_ = V_DS_ and V_GS_ > V_DS_ stress condition on p-type GAA NS, which contributes from gradually dominated of NBTI mechanism.

Curvature part occupies more percentage on NS when T_si_ becomes thinner and more severe corner field crowding effect exists at scaled diameters of the curved region [24]. Enhanced HCD is expected at GAA NS transistors with thinner T_si_, which was observed and reported in [36]. 

Transportation surface is critical to transistor mobility. Hole mobility was reported to be higher in channels with (110) transport orientation, which was widely used in manufactures as one of the key elements to boost p-type transistor performance [32,39,40,41]. Surface orientation impact to HCD of GAA NS transistors was reported and discussed in [36]. HCD was observed more serious in p-type GAA NS transistors with (110) channel surface orientation vs. the ones with (100) channel surface orientation, which attributes to more defects generated during stress in GAA NS transistors with (110) channel surface orientation. HCD was found comparable on n-type GAA NS transistors with (100) and (110) channel surface orientations, which was explained by performance delta from transistors with different surface orientations. Details will be discussed in next section (Section 2.5) regarding HCD and performance trade-off.

### 2.5. Inner Spacer and Junction Dependent

Gate to epitaxy source/drain distance and junction are two of the important elements impacting HCD on both planar FETs and FinFETs, as well as the impact on GAA NS transistors [42,43,44]. Inner spacer, as one of the key specific components of GAA NS transistors, plays crucial role in HCD together with junction between epitaxy source/drain and channel. A limited study was conducted in this topic on GAA NS transistors.

**Figure 14:**
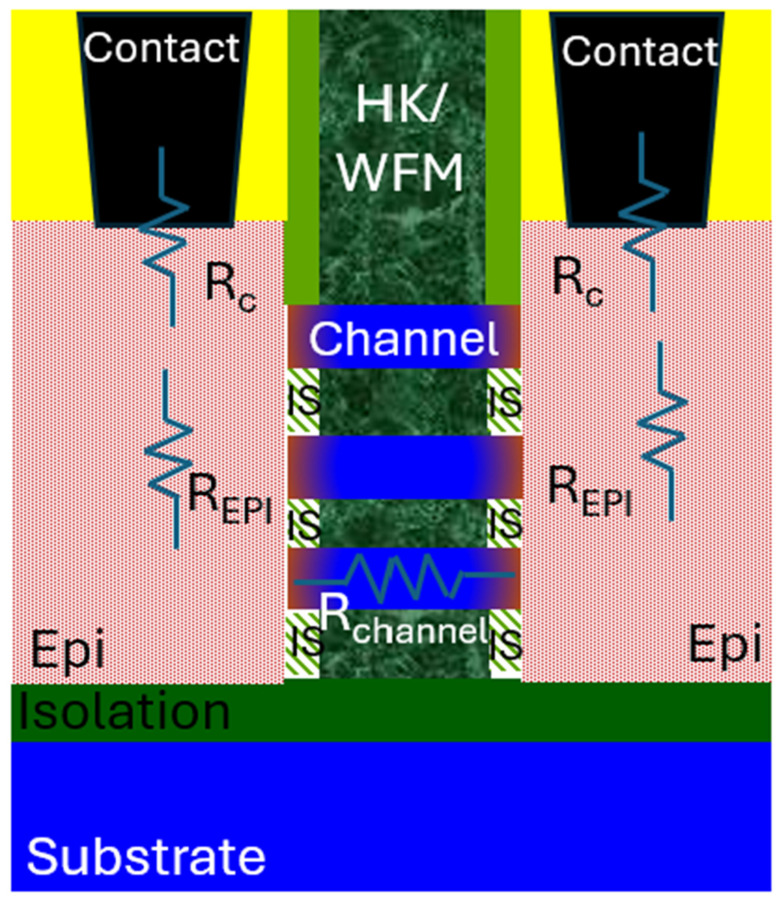
Schematic diagram of GAA NS transistors for self-aligned substrate isolated GAA NS. Transistor resistance components: R_C_, R_EPI_ and R_channel_ are marked in the Figure.

Figure 14 shows schematic diagram of reference GAA NS transistors with self-aligned substrate isolation. Transistor resistance components, R_C_ (metal to source/drain resistance), R_EPI_ (epitaxy source/drain resistance) and R_channel_ (channel resistance) are marked in the Figure Reducing the resistance components play an important factor in improving performance and are also critical to HCD.

**Figure 15:**
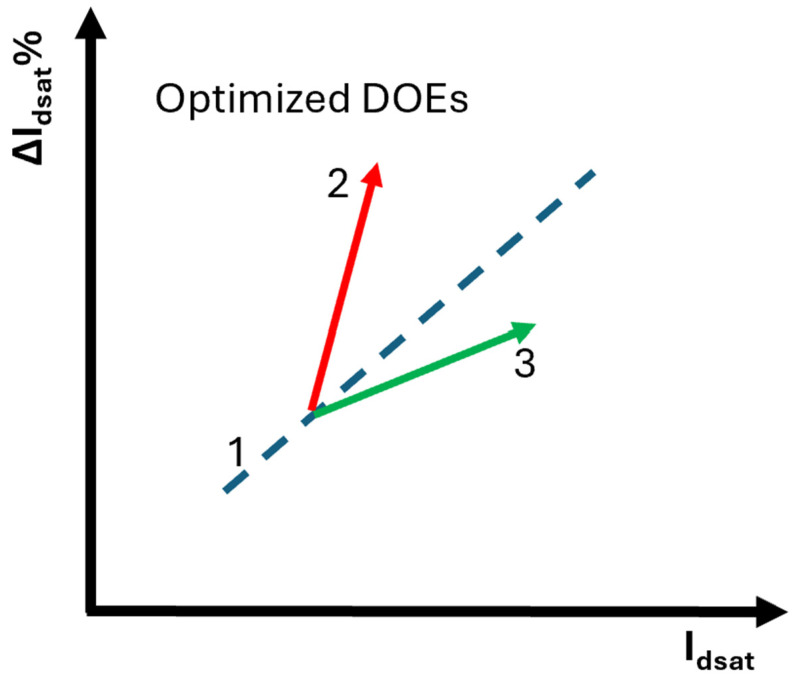
HCI EOL and. I_dsat_ trade-off trend. Design and process caused performance boost or I_dsat_ increasing could correspond to different slope in the trend.

HCD EOL typically degrades with I_dsat_ increasing. Design and process caused performance boost corresponding to different HCD-performance trade-off trend is plotted in Figure 15. Trend mark “1” (blue color) can result from, but not limited to, channel length scaling. Trend mark “2” (red color) can result from, but not limited to, worse junction profile and higher gate oxide traps density. Trend mark “3” (green color) can be attribute to, but not limited to, junction profile optimization and gate oxide quality improvement [19,21]. It’s worth to point out that it’s important to decouple resistance components, R_channel_, R_C_ and R_EPI_, in performance improvement to better understand the performance boost and HCD trade-off since voltage drop along R_channel_ is one of the critical parameters impacting HCD reliability.

Inner spacer thickness impact and junction optimization to HCD on n-type GAA NS transistors with self-aligned substrate isolation was reported in [21]. In the article, 3D schematics of self-aligned substrate isolated GAA NS transistors were plotted together with simplified process on IS thickness and junction DOEs. Junction profile of GAA NS transistors with reference inner spacer, thinner inner spacer thickness and junction DOEs was simulated and discussed. The study indicated more dopants overlap and abrupt junction existing in GAA NS transistors with junction DOE. HCD and performance boost on GAA NS transistors with both DOEs were evaluated and discussed in the paper. GAA NS transistors with junction DOE show promising results on HCD and performance trade-off comparing with the one with IS thickness DOE. HCD was reported worse in GAA NS transistors with inner spacer DOE in the trade-off trend, indicating additional traps introduced during stress on GAA NS transistors with IS thickness reduction DOE. The study suggested IS quality improvement is required for further scaling.

**Figure 16:**
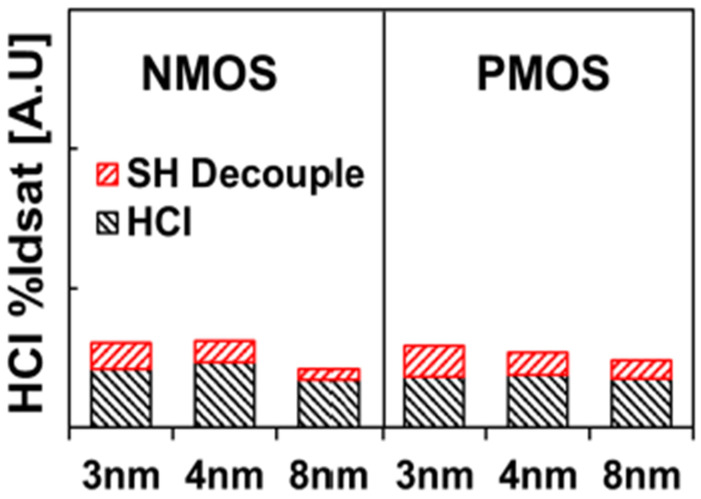
Normalized HCI I_dsat_% for different technology node. 3 nm is the technology node with GAA NS architecture while others are technology nodes from FinFET structure [15]. Reprinted/adapted with permission from IEEE Proceedings of the 2023 International Reliability Physics Symposium.

Kim et al. reported HCD study on 3 nm GAA architecture and compared with prior technology. Transistor architecture was illustrated in the article and no inner spacer was observed between source/drain and HK/WFM. Figure 16 shows normalized HCI I_dsat_% for different technology node. 3 nm is the novel technology node with GAA NS architecture while others are technology nodes with FinFET structure. The author concluded that HCD is comparable on GAA NS transistors versus the one of their prior technologies [15]. However, it’s worth to point out that parallel capacitance on GAA NS transistors with non-inner spacer structure may bring more concern in AC functional circuits, which results in DC and AC performance trade-off and brings hot topic on DC and AC performance balance.

### 2.6. Dielectric Wall Effect (Fork Sheet Specific)

**Figure 17:**
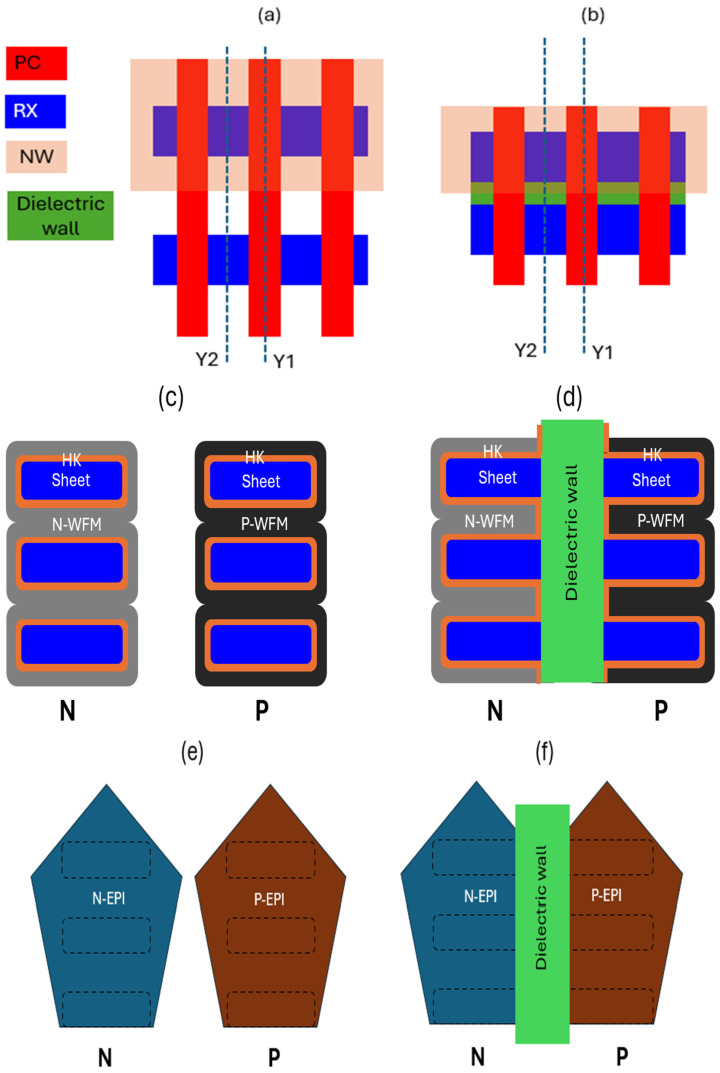
(**a**,**c**,**e**) Top-down layout and cross-section schematic diagram of standard gate all around structure in cross sheet/along PC (Y1) direction and cross sheet/along epitaxy (Y2) direction. (**b**,**d**,**f**) Top-down layout and cross-section schematic diagram of fork sheet in cross sheet/along PC (Y1) direction and cross sheet/along epitaxy (Y2) direction. Dielectric wall exists between N and P type RX in (**d**,**f**).

Fork sheet, a 3D architecture of sacrificing one side gate control to achieving further scaling down comparing with standard gate all around structure, requires a specific module as called dielectric wall be form as reported in [45]. Figure 17a shows standard GAA NS transistor layout with key layers such as active sheet (RX), Gate (PC) and N-Well (NW). Figure 17c,e show cross-section diagrams of standard gate all around structure in cross sheet/along PC(Y1) direction and cross sheet/along epitaxy direction(Y2), respectively. Figure 17b shows layout of fork sheet transistor with key layers and structure such as RX, PC, NW and dielectric wall. Figure 17d,f show cross-section diagrams of fork sheet structure cross sheet/along PC (Y1) direction and cross sheet/along epitaxy direction(Y2), respectively. Compared with fully wrapped HK/WFM nanosheet in Figure 17c, side wall of fork sheet was attached with dielectric film in fork sheet structure, as shown in Figure 17d. In Figure 17f, self-aligned N and P type epitaxy sources (drains) are isolated by rectangular dielectric wall while no similar dielectric wall exists between N and P type epitaxy sources (drains) in Figure 17e. The lack of fully wrap around gates from fork sheet architecture, decreases short channel control, the direct contact of channel to the SiN based dielectric wall also brings reliability concerns. Researchers have worked on process optimization and reliability validation to evaluate impact of dielectric wall in fork sheet [24,44].

**Figure 18:**
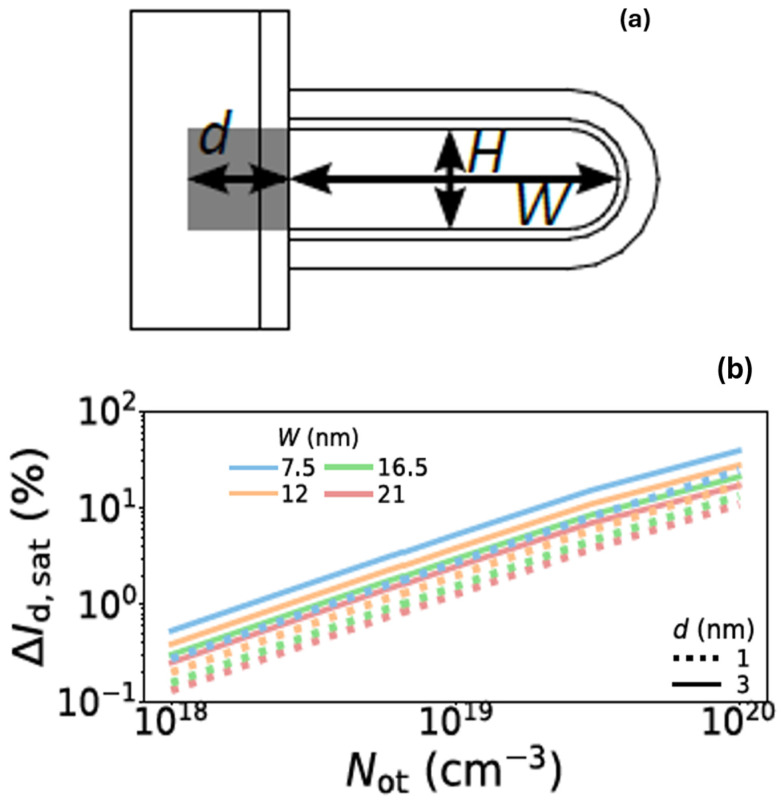
(**a**) Schematic diagram of dielectric wall with one fork sheet; (**b**) HCD from various fork sheet width and different charge depths to the dielectric wall [24]. Reprinted/adapted with permission from IEEE Proceedings of the 2022 International Reliability Physics Symposium.

Figure 18a shows schematic diagram of dielectric wall with one fork sheet. Figure 18b shows HCD from various fork sheet width and different charge depth to the dielectric wall. It’s found that HCD aggravated with more oxide charges from dielectric wall. When sheet width decreasing, larger part of the channel is close to the charges in the dielectric wall, HCD becomes worse [24]. It’s also noted by the author that there is limited contribution to HCD from less defective dielectric wall. The dielectric wall impact to HCD is negligible with careful integration and high-quality dielectric wall formed.

### 2.7. Body Isolation Effect

Body-Isolation (BDI) scheme was proposed by Zhang et al. in [46], which can provide significant benefit comparing with the conventional punch-through-stopper (PTS) process. Full BDI scheme introduces a dielectric layer below both the source/drain and gate regions to reduce leakage and improve power-performance.

**Figure 19:**
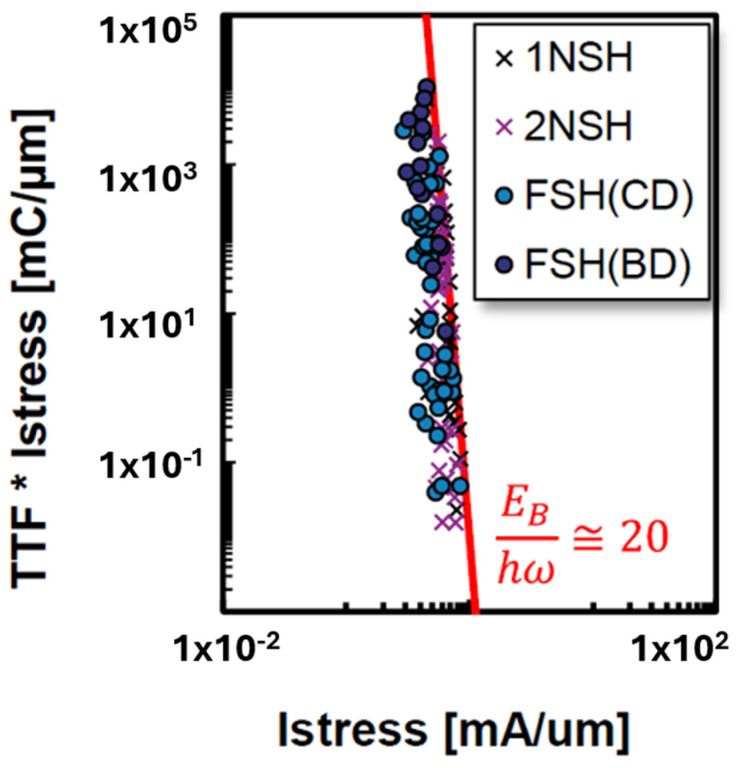
Time to failure at 10% I_DSAT_ × I_STRESS_ vs. I_STRESS_ for multi-vibrational excitation (MVE) evaluation on p-type fork sheet transistors [48]. Reprinted/adapted with permission from IEEE Proceedings of the 2023 International Reliability Physics Symposium.

Mertens et al. embedded body isolation solutions to fork sheet in [47] and Bury et al. performed HCD studies on traditional fork sheet comparing to fork sheet with BDI in [48]. Figure 19 shows time to failure at 10% I_DSAT_×I_STRESS_ vs. I_STRESS_ for multi-vibrational excitation (MVE) evaluation on p-type fork sheet transistors. No special trapping effect was observed in p-type BDI fork sheet as concluded by the authors.

### 2.8. HCD and Self-Heating

**Figure 20:**
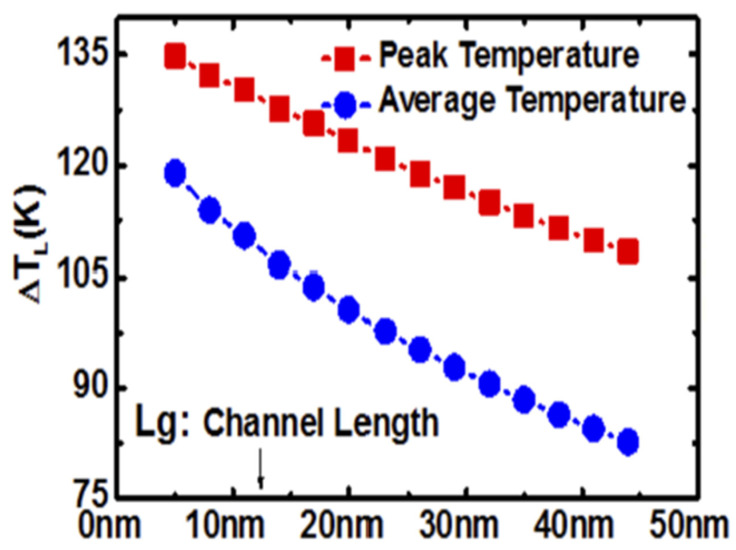
Temperature rising from self-heating with channel length scaling on GAA NS Transistors [53]. Reprinted/adapted with permission from 2018 International Symposium on VLSI Technology, Systems and Application (VLSI-TSA).

Despite the superior gate control and transistor performance, the vertically stacked GAA structure results in increased self-heating concern. The introduction of 3D gate-all-around architecture makes the heat in the channel hard to dissipate, leading to a noticeable self-heating and thermal concern, which may significantly impact accurate projection of transistor reliability [21,35,46,47,48,49,50,51,52,53,54,55]. Figure 20 shows temperature rising on GAA NS transistors with different channel length, reported by Cai et al. in [53]. It observed that temperature rising from the channel could increase as high as 100 K and more when scaling the channel length to sub 10 nm.

**Figure 21:**
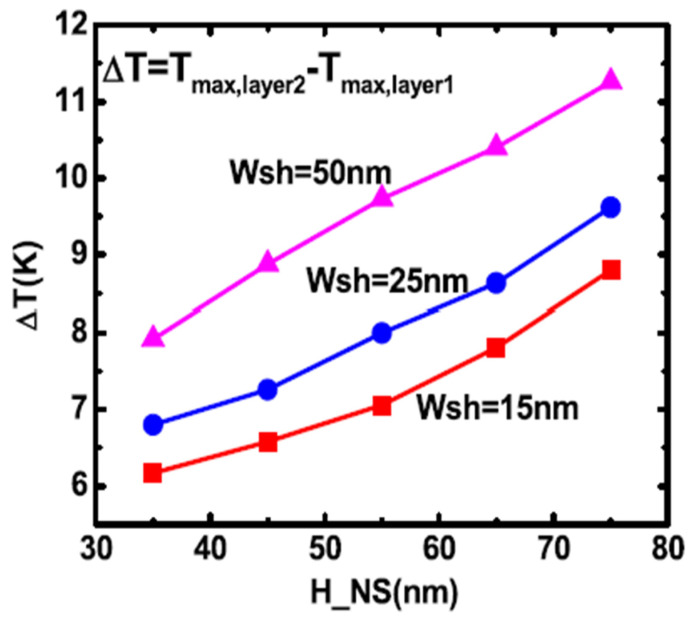
Peak temperature (ΔT) between different layers from various W_sh_ and stacked nanosheets height [54]. Reprinted/adapted with permission from 2018 IEEE TRANSACTIONS ON ELECTRON DEVICES.

Layout dependent self-heating impact on FinFET was studied and reported by Mittl et al. in [52]. More serous self-heating effects was observed in dense design of FinFet technology. Layout effects of self-heating impact on GAA NS transistors were further studied and reported by Cai et al. in [54]. Self-heating impact was found aggravated in wider sheet width. Figure 21 shows peak temperature of various W_si_ and stacked nanosheet height. It’s observed that the wider sheet width, more temperature delta between different layers. Higher sheet height also induced more temperature delta from the study.

**Figure 22:**
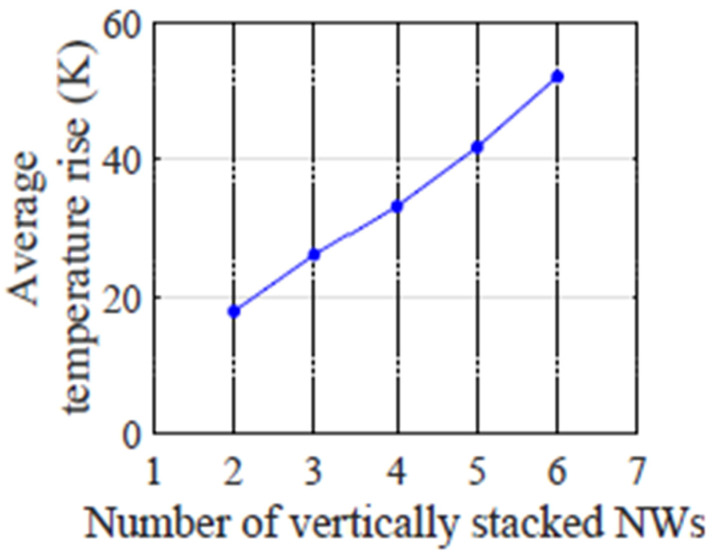
Rising temperature vs. vertical stacked GAA NWs numbers [55]. Reprinted/adapted with permission from 20th International Symposium on Quality Electronic Design, 2019.

Self-heating as a function of the number of vertical stacked nanowires was also studied and reported in [55]. Figure 22 shows rising temperature vs. number of vertically stacked GAA nanowires transistors. It’s observed that with more NS numbers stacked on the transistors, it becomes more difficult for heat to dissipate, contribute to higher rising temperatures.

Channel temperature is higher than chuck temperature from self-heating effect in transistor operation and is even higher under HCI stress. Voltage Accelerate Factor (VAF) and Activation Energy (E_A_) of HCD on GAA NS transistors, which are two important modeling parameters of HCD, are lack of exactitude without self-heating correction. VAF and E_A_ of HCD on GAA NS transistors were studied, corrected for self-heating and compared with their raw number in [21]. It’s reported that after self-heating correction, VAF was found decreased, attributing from more self-heating impact at high power. Activation Energy (E_A_) was also observed decrease post self-heating correction, which attributed from self-heating correction at different temperature. 

Since Self-heating effects is aggravated in vertical stacked GAA NS transistors comparing with prior generation FinFETs, it is critical to make accurate reliability projection with precise thermal modeling and characterization methodology. Numerous studies from academic and industrial sources have reported various characterization methodology, modeling and designs to decouple self-heating impact to HCD [55,56,57,58,59]. Self-heating effect on HCD EOL ∆I_dsat_% was corrected and reported with 10%~35% correction from different DOEs, process and performance [2,21]. The topic of self-heating correction methodology on HCD is still open to exploration and solution.

## 3. Challenges and Future Outlook

This paper discussed the HCD impact in the gate-all-around nanosheet transistor technology, consolidating some of the pioneering work in the field over the last couple years. HCD, as one of the key reliability mechanisms still presents tremendous challenges to the technology. The challenges may be broadly categorized into four areas: gate length further shrink, geometry, corner effect and self-heating.

As one of the key advantages of GAA nanosheet, flexible sheet width is allowed in design. Geometry dependance of nanosheets is critical to reliability. As sheet width further shrink during scaling down especially in high density circuit design, sheet corners could dominate HCD. T_si_ and curvature range could be different at various sheet width from channel release, which require high selective indent process to improve the uniformity. Inner spacer thickness engineering requires robust dielectric material to mitigate HCD effect for further scaling down and junction optimization is one of the effective elements to modulate HCD and performance trade-off [21]. Another aspect of the challenges is the device variability from, but not limited to, gate length, inner spacer thickness scaling down, are critical to overall or full wafer HCD.

Self-heating effects in GAA NS transistors result in significant thermal concern, contribute to device performance and reliability degradation [21,44,45,50,51]. Self-heating correction has been performed and compared with prior technology [15,21]. Many designs and studies have been explored and developed for self-heating correction and accurate HCI EOL projection [21,36,60,61], which is still open to exploration and solution.

Finally, direct backside contact, as one of the key components in backside distribution net, moving one of the contacts to backside [62,63,64,65], providing space on the front-side for routing flexibility [66,67,68], has not been discussed here. The new scheme has been proved exists more self-heating concern and reliability challenges on HCD, brings tremendous opportunities of innovations and characterization methodologies development on technology qualification.

## Data Availability

No new data were created in this study.
